# Complexity Collapse, Fluctuating Synchrony, and Transient Chaos in Neural Networks With Delay Clusters

**DOI:** 10.3389/fnsys.2021.720744

**Published:** 2021-11-19

**Authors:** S. Kamyar Tavakoli, André Longtin

**Affiliations:** Department of Physics and Centre for Neural Dynamics, University of Ottawa, Ottawa, ON, Canada

**Keywords:** dynamical system, transient chaos, delayed differential equation, synchrony, neural network, neural dynamics

## Abstract

Neural circuits operate with delays over a range of time scales, from a few milliseconds in recurrent local circuitry to tens of milliseconds or more for communication between populations. Modeling usually incorporates single fixed delays, meant to represent the mean conduction delay between neurons making up the circuit. We explore conditions under which the inclusion of more delays in a high-dimensional chaotic neural network leads to a reduction in dynamical complexity, a phenomenon recently described as multi-delay complexity collapse (CC) in delay-differential equations with one to three variables. We consider a recurrent local network of 80% excitatory and 20% inhibitory rate model neurons with 10% connection probability. An increase in the width of the distribution of local delays, even to unrealistically large values, does not cause CC, nor does adding more local delays. Interestingly, multiple small local delays can cause CC provided there is a moderate global delayed inhibitory feedback and random initial conditions. CC then occurs through the settling of transient chaos onto a limit cycle. In this regime, there is a form of noise-induced order in which the mean activity variance decreases as the noise increases and disrupts the synchrony. Another novel form of CC is seen where global delayed feedback causes “dropouts,” i.e., epochs of low firing rate network synchrony. Their alternation with epochs of higher firing rate asynchrony closely follows Poisson statistics. Such dropouts are promoted by larger global feedback strength and delay. Finally, periodic driving of the chaotic regime with global feedback can cause CC; the extinction of chaos can outlast the forcing, sometimes permanently. Our results suggest a wealth of phenomena that remain to be discovered in networks with clusters of delays.

## 1. Introduction

Biological neural networks can involve delays below the millisecond time scale up to several tens of milliseconds (Madadi Asl et al., 2018). A wide array of delays are involved in inter-areal communication (Deco et al., 2009). A redundancy cancellation circuit in the cerebellum of the weakly fish involves delay distributions between 10 and 70 ms (Bol et al., 2011). Local circuitry also involves delays, which are often neglected in modeling studies due to the added dynamical complexity they bring to the problem. But they have been shown to promote oscillations (Belair et al., 1996; Brunel and Hakim, 1999; Bimbard et al., 2016), and play important roles in synchronization phenomenon (Coombes and Laing, 2009) and learning phenomenon (Gerstner et al., 1996). They are of course omnipresent in large scale neural control systems where they can reach many hundreds of milliseconds, e.g., in reflex arcs (Longtin et al., 1990).

What are the dynamical consequences of the existence of multiple delays, either centered around a single mean delay, or clustered into different groups? There is widespread belief that systems with many delays can be treated as ones with a single distribution of delays, i.e., a delay-differential equation with discrete delays can be replaced by an integro-differential equation with a suitably chosen delay or memory kernel. Accordingly, the presence of many delays with a sufficiently broad distribution should decrease the dynamical complexity (Longtin, 1990; Jirsa and Ding, 2004; Eurich et al., 2005; Tavakoli and Longtin, 2020).

Recently it has been shown, using numerical experiments of simple model physical systems along with a novel Lyapunov spectrum estimation method for multi-delay non-linear systems, that this complexity reduction can happen quite abruptly, and therefore be more aptly named complexity collapse (Tavakoli and Longtin, 2020). The effect has been investigated by adding delays to standard one-delay systems in one variable, such as the Mackey-Glass equation and the electro-optic model, or the three-variable Lang-Kobayashi laser model. Our work here raises and provides first answers to the question of whether this multi-delay complexity collapse (MDCC) can occur in chaotic neural networks with multiple neurons, i.e., with many state variables.

Note that we are distinguishing here between the number of state variables that describe the time-varying quantities in these models, and the infinite number of variables that relate to the delay *per se*; all differential-delay systems are infinite-dimensional by definition, regardless of the number of delays. Beyond this distinction, it therefore remains to be seen how a cluster of delays around some mean delay affects the chaotic properties of a neural network, and whether additional clusters further cause increases or decreases in dynamical complexity. While our previous study allowed for a more precise diagnostic of attractor properties, using permutation entropy and Lyapunov spectrum estimation, here the large number of state variables (around 1,000) make such computations prohibitively expensive. We thus resort to other simpler metrics that focus on the time-dependent mean and standard deviation of the activity variable averaged across the network.

Of particular interest to us is the question of under which conditions and with respect to which phenomena do delays matter in realistic neural systems. The particular aspect of this question that we focus on is the distribution of discrete delays. Such delays, even acting alone, are notorious for causing simple oscillations and, with the right shape and strength of non-linearities, chaotic fluctuations; yet distributed delays are known to counteract some effects of non-linearity (Longtin, 1990; Herrmann et al., 2016). At which point should one think in terms of continuous delay distributions, and what is expected in the remaining vast domain between single and distributed delays? And how are these issues at play in chaotic neural nets? One expects that bifurcations can occur, but also novel forms of multistability and susceptibility to rhythms impinging from other brain areas. Such effects are indeed highlighted in the results presented below, along with their robustness to noise.

**Figure 1:**
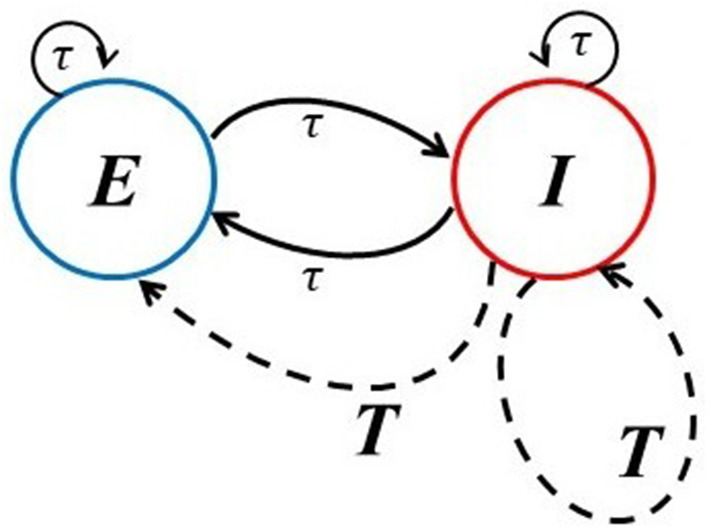
Network architectures. The solid lines provide the schematic of the basic excitatory-inhibitory (EI) network in which the connections can have multiple smaller delays (denoted here by τ) characteristic of local circuitry. The dashed lines account for an extra global inhibitory feedback with longer delay *T* from population I to itself and to the E population.

In section 2, we introduce the model of interest, namely, a standard 80/20 excitatory-inhibitory (EI) model that has often been used to mimic the cortex. It has local delays between the E and I cells, but can also account for a global delayed inhibitory feedback to both populations with a larger delay (see Figure 1, dashed lines). This global feedback mimics a longer route for inhibition that possibly involves other populations that are not explicitly modeled. It is considered here because the complexity collapse phenomenon (CC) does not occur in the EI network on its own, but does in this slightly more complex dynamical system with two delay clusters. In the section 3, we thus first probe how a single delay within and between these sub-populations affect the dynamics. In this network, neurons behave in a much more complex manner as the time delay becomes smaller. Next, we examine the activity and complexity of dynamics generated by neurons under the influence of the global inhibitory feedback term. We will present a novel form of behavior that is reminiscent of a chimera (Larger et al., 2013; Majhi et al., 2019; Sawicki et al., 2019), but with space replaced by time. In order words, we report an alternation of asynchronous and synchronous epochs which seem to follow Poisson statistics. We further show a paradoxical effect in which the activity fluctuations are more constrained the higher the noise is, which is a form of noise-induced order (Matsumoto and Tsuda, 1983). As a consequence of the inclusion of the additive noise with sufficiently large intensity, synchronous activity can be suppressed.

We further provide preliminary observations of the effect of periodic driving of the excitatory sub-population during synchronous epochs, finding that it can alter the dynamics of the whole network. Post-stimulation dynamics can be unpredictable, leading either to transient high-frequency oscillations followed by a return to chaotic dynamics with synchronous epochs, or to CC with periodic behavior. The possibility of observing CC in the presence of the global inhibitory feedback and external stimuli led us to finally study the dynamics of these sub-networks in the presence of multiple local time delays. The non-linear characteristic of this network prevents CC in the absence of the global inhibitory delayed feedback. However, this non-linearity is seemingly weaker when distributed delays in the local recurrent EI circuitry co-occur with a global delay. For a larger width of the distribution of delays, the transient chaos is replaced by a simple oscillation.

Note that, for the sake of brevity, none of the phenomena reported here are analyzed individually in great detail. We have rather opted for a presentation of a few novel effects related to CC that will hopefully guide future studies; all our results are linked by the existence of multiple delays in various clustered configurations.

## 2. Models and Methods

We consider an excitatory and an inhibitory sub-network of rate model neurons, each coupled within itself and to the other sub-network. The architecture corresponding to this network is shown in Figure 1. The potential of an excitatory neuron is designated as *u*, and an inhibitory neuron as *v*. A similar model without local delay and global inhibitory feedback delay has been studied in Rich et al. (2020). In parts of our work, we go beyond this model by assuming that each of these sub-networks is also affected by global delayed inhibitory feedback from the inhibitory cells, with a global feedback strength κ; this global feedback delay is made longer than the local recurrent feedback delay. The delayed feedback aspects of our model are similar to those in Herrmann et al. (2016) and Hutt et al. (2016). The dynamical equations for the potential of each unit in the network are:


(1a) $$\begin{array}{l} \alpha_{e}^{-1}\frac{du_{j}}{dt}=-u_{j}+\frac{1}{n_{e}}\frac{1}{M}\overset{M}{\underset{l=1}{\sum }}\overset{N_{e}}{\underset{k=1}{\sum }}w_{jk}^{ee}\phi \left(u_{k}\left(t-\tau_{l}\right)\right)\\ +\frac{1}{n_{i}}\frac{1}{M}\overset{M}{\underset{l=1}{\sum }}\overset{N_{i}}{\underset{k=1}{\sum }}w_{jk}^{ie}\phi \left(v_{k}\left(t-\tau_{l}\right)\right)+\frac{\kappa }{N_{i}}\overset{N_{i}}{\underset{k=1}{\sum }}\phi \left(v_{k}\left(t-T\right)\right)\\ +\sigma \xi_{E}+S\left(t\right) \end{array}$$

(1b) $$\begin{array}{l} \alpha_{i}^{-1}\frac{dv_{j}}{dt}=-v_{j}+\frac{1}{n_{e}}\frac{1}{M}\overset{M}{\underset{l=1}{\sum }}\overset{N_{e}}{\underset{k=1}{\sum }}w_{jk}^{ei}\phi \left(u_{k}\left(t-\tau_{l}\right)\right)\\ +\frac{1}{n_{i}}\frac{1}{M}\overset{M}{\underset{l=1}{\sum }}\overset{N_{i}}{\underset{k=1}{\sum }}w_{jk}^{ii}\phi \left(v_{k}\left(t-\tau_{l}\right)\right)+\frac{\kappa }{N_{i}}\overset{N_{i}}{\underset{k=1}{\sum }}\phi \left(v_{k}\left(t-T\right)\right)\\ +\sigma \xi_{I} \end{array}$$

where the τ_*l*_'s are the local conduction delays which may all be the same, or be taken from a discrete probability density. ξ_*E,I*_(*t*) denote Gaussian white noises, chosen for simplicity here as having the same strength $\sigma =\sqrt{2D}$ with < ξ_*E,I*_(*t*) > = 0 and $<\xi_{i}\left(t\right)\xi_{j}\left(t^{\prime }\right)>=\delta_{ij}\delta \left(t-t^{\prime }\right)$.

The firing rate function ϕ follows a sigmoidal function defined as:


(2) $$\begin{array}{l} \phi \left(u\right)=\frac{1}{1+e^{-\beta u}}. \end{array}$$

All parameters are described in Table 1. Some of our last results consider the effect of a periodic input *S*(*t*) of different frequencies to the excitatory population. In some of our simulations below, we will consider multiple delays chosen from a discrete density. This means that each unit is connected to all other units with these multiple delays.

**Table 1:** Parameters of the two-population model.

**Symbol**	**Definition A**	**Value**
*N*_*e*_	Number of excitatory units	800
*N*_*i*_	Number of inhibitory units	200
α_*i*_	Dendritic rate constant—inhibitory	200 Hz
α_*e*_	Dendritic rate constant—excitatory	100 Hz
β	Response function gain	100
*n*_*e*_	Number of excitatory connections for each neuron	80
*n*_*i*_	Number of inhibitory connections for each neuron	20
*w* ^*ee*^	*e*→*e* Synaptic connection strength	15
*w* ^*ei*^	*e*→*i* Synaptic connection strength	15
*w* ^*ie*^	*i*→*e* Synaptic connection strength	−15.375
*w* ^*ii*^	*i*→*i* Synaptic connection strength	−15.375
κ	Global feedback strength	Variable
*M*	Number of delays	Variable
*D*	Intrinsic noise level	Variable
*dt*	Integration timestep	0.1 ms

**Figure 2:**
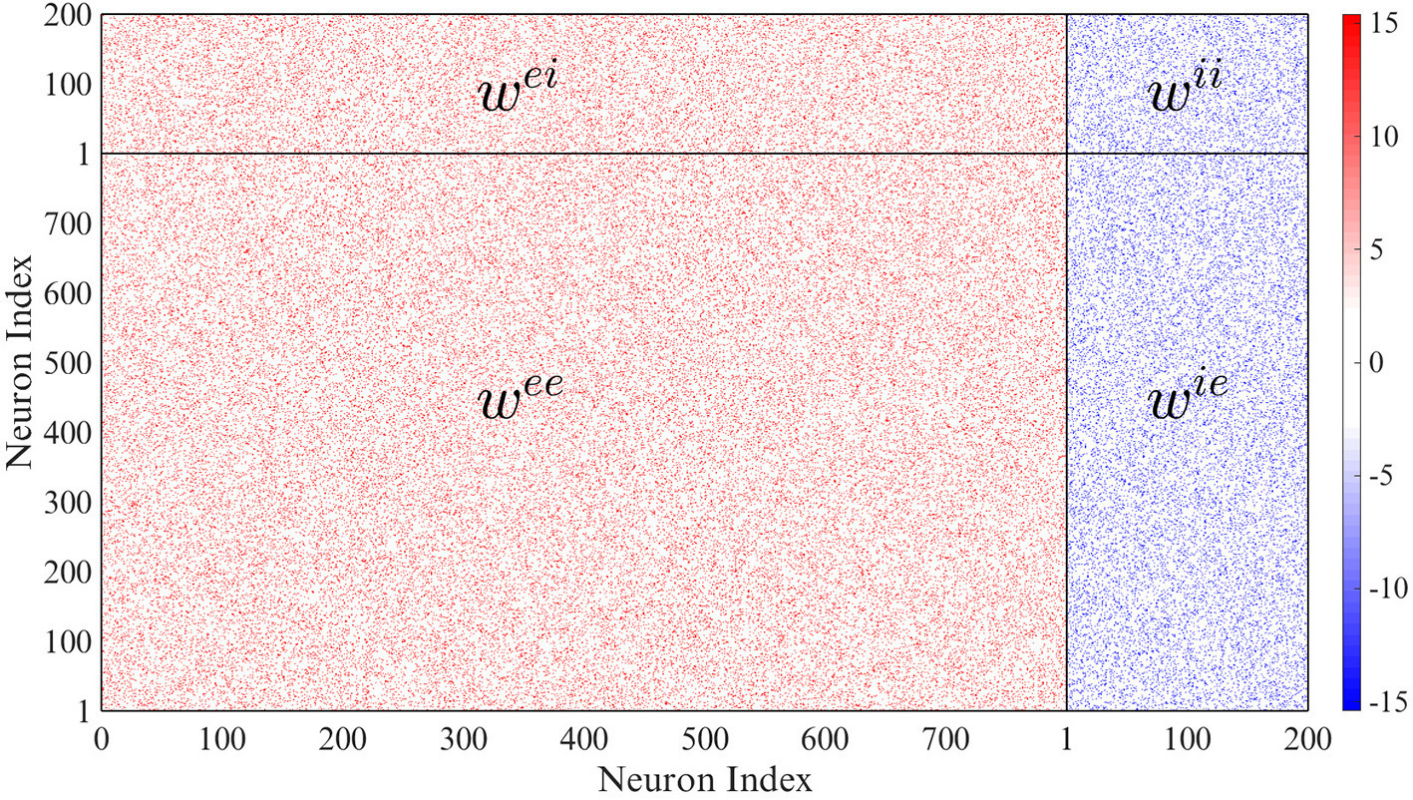
Network connectivity. Only 10 percent of the weights are non-zero.

We assume that there are *N*_*e*_ = 800 excitatory units and *N*_*i*_ = 200 inhibitory units in the whole network, and that the probability of connection of any two neurons is 10%. Thus each neuron is connected on average to 100 other neurons. The weight matrix can be seen in Figure 2 in which the excitatory connection weights are fixed at 15 and the inhibitory weights at −15.375 (the mean of the network and mean of the non-zero connections in the network are approximately zero). The initial conditions are picked randomly from a Gaussian distribution with zero mean and unit variance. This choice of values gives a slightly unbalanced network: there are 4 times more excitatory neurons than inhibitory neurons, but the inhibitory weight divided by the number of inhibitory connections ($\frac{w^{ii}}{n_{i}}=\frac{w^{ie}}{n_{i}}$) is 4.1 times the excitatory weight divided by the number of excitatory connections ($\frac{w^{ei}}{n_{e}}=\frac{w^{ee}}{n_{e}}$). We have checked that the phenomena reported here are robust in the sense that they are qualitatively the same when the network is set up with similar weight ratios, and in particular for the balanced case where the ratio is equal to 4, i.e., with *w*^*ii*^ = *w*^*ie*^ = −15. The results below are also qualitatively similar for the case where elements of the weight matrices are picked randomly from Gaussian distributions such that the mean of the excitatory neurons is 15 and the mean of the inhibitory neurons is −15.375. Also, in the absence of any delays, our network is in a chaotic state, as it is with small local delays in the absence of global feedback and noise. In the thermodynamic limit, the complexity of the dynamics decreases; however, complex dynamics can still be observed provided that smaller delay values are used (at least for the parameters *N* = 10000, *n*_*e*_ = 800, and *n*_*i*_ = 200 that we tested).

## 3. Results

**Figure 3:**
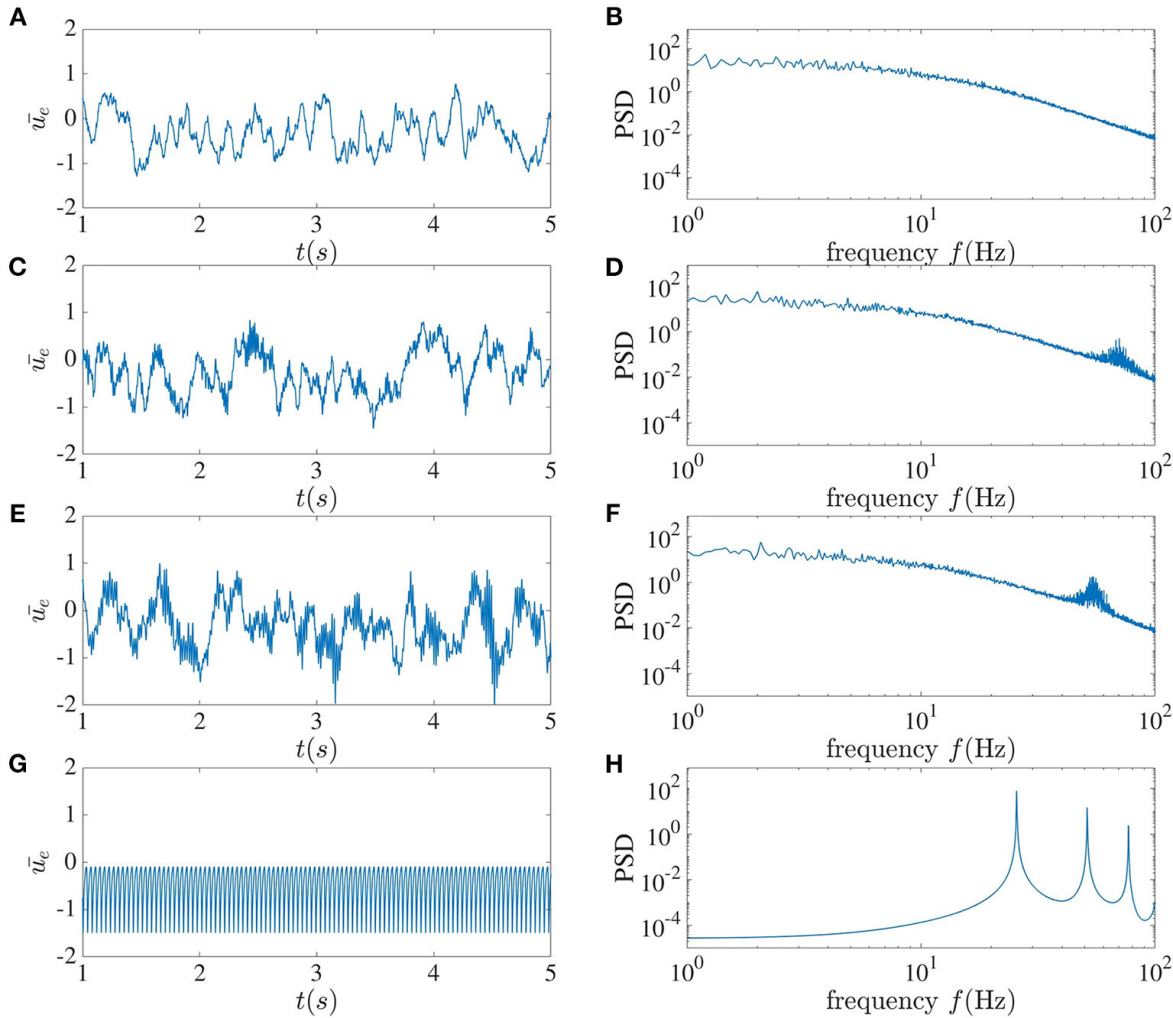
Local dynamics alone. The left column shows the mean activity of the excitatory sub-network, and the right column plots the corresponding power spectrum averaged over the activities in this sub-network. From top to bottom, the local time delay corresponds to **(A,B)** 2 ms, **(C,D)** 5 ms, **(E,F)** 7 ms, and **(G,H)** 10 ms.

The mean of the activity of the excitatory sub-network for different time delays between interacting neurons can be seen in Figure 3. For τ = 2 ms, chaotic behavior can be observed, with no clear peak in the power spectrum, which in fact has power-law characteristics. As the time delay increases to 5 ms, a peak arises in the power spectrum at 70 Hz. This peak further shifts toward the lower frequency of 55 Hz as the delay increases. When the time delay between neurons in the local recurrent circuitry is increased to 10 ms, chaotic dynamics can no longer be seen, and harmonics appear in the power spectrum at integer multiples of 25.6 Hz. For this latter case, when the dynamic is affected by noise, one can use the mean-field method introduced in Hutt et al. (2016) to study the dynamical property of the network. It can be concluded that in this system, a larger delay leads to more coherence between the neurons' activities. We should mention that when we increase connection numbers *n*_*e*_ to 800 and *n*_*i*_ to 200, and the total number of units to 10, 000 for this set of parameters, the dynamical behavior becomes simpler; but chaotic behavior can still be achieved for smaller time delays.

**Figure 4:**
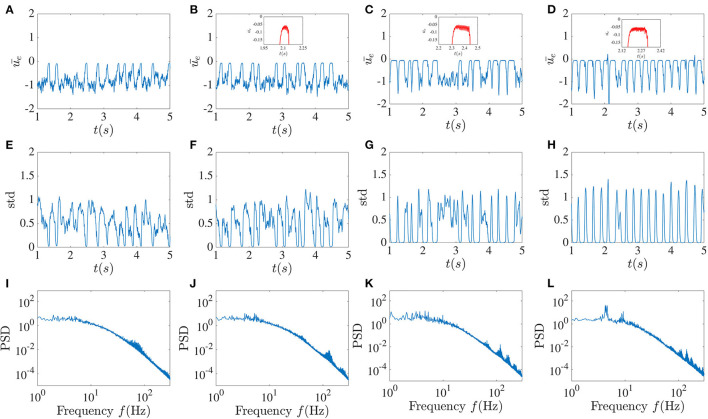
Higher global feedback delay causes activity dropouts. Mean of the excitatory sub-network activity **(A–D)**, the standard deviation of the activities of its units **(E–H)**, and the power spectrum averaged over the units in the excitatory sub-network **(I–L)** in the presence of global inhibitory feedback with fixed strength κ = −5 without noise. From left to right, the global feedback time delay *T* equals 5, 10, 20, and 30 ms. The pulse-like epochs in the solution correspond to “activity dropouts” where the sub-network is synchronized with a low firing probability. Paradoxically, between these dropouts, the time-dependent mean activity is lower but its time-dependent fluctuations are stronger. Insets in the figures in the first row show the high-frequency low-amplitude oscillations that occur during the dropouts. The more regular pulsing in **(D)** is associated with a low frequency peak and its harmonics riding on top of the broadband background.

In the next step, we examined how delayed global inhibitory feedback from inhibitory units influences network dynamics. In Figure 4, the dynamical behavior of the excitatory network for different global feedback time delays and the smaller fixed local time delay is shown. Without local delayed interactions, the activity is a regular oscillation as is expected from purely inhibitory networks with delay. Here we took the local time delay τ = 2 ms and did the simulation for the fixed value of the global feedback coefficient κ = −5 and variable global feedback time delay *T*. The global feedback tends to align the dynamical behavior of all units together, while the influence of the local time delays leads to chaotic fluctuations.

The existence of the global feedback, along with the small local delay, causes the appearance of a pattern of very low activity punctuated by random, sudden and brief jumps to larger values. We call these behaviors “dropout activities.” They can be characterized by the time-dependent standard deviation (SD) of the activity across the units in the excitatory sub-network (Figure 4, middle panels). A stronger global feedback tends to weaken the chaotic nature of the units. Each time that the dynamics enter the state of deficient firing rate activity, the standard deviation becomes very close to zero, meaning that the whole network is highly synchronized in this low activity state. Below we will see the paradoxical implications of this behavior for spiking activity using a spiking rule on top of the activities; spikes will be associated with the state of lower mean activity because they are caused by strong fluctuations, i.e., it is a fluctuation-driven spiking regime.

We can gain more insight by looking at the mean of the power spectrum of the excitatory sub-network's activity. As a result of increasing the global feedback time delay, we can observe that the peak around the 3–8 Hz low-frequency component becomes sharper, and thus that there is enhanced more regular low-frequency activity, a feature that stands out from the time series. Furthermore, it can be seen in the insets that these dropout activities are associated with high-frequency oscillations with very low amplitude, which are also evident in the power spectrum. As the global feedback time delay increases, the higher frequency components become more prominent, such that for *T* = 30 ms there are more high-frequency peaks that are positioned approximately 30 − 40 Hz from each other.

**Figure 5:**
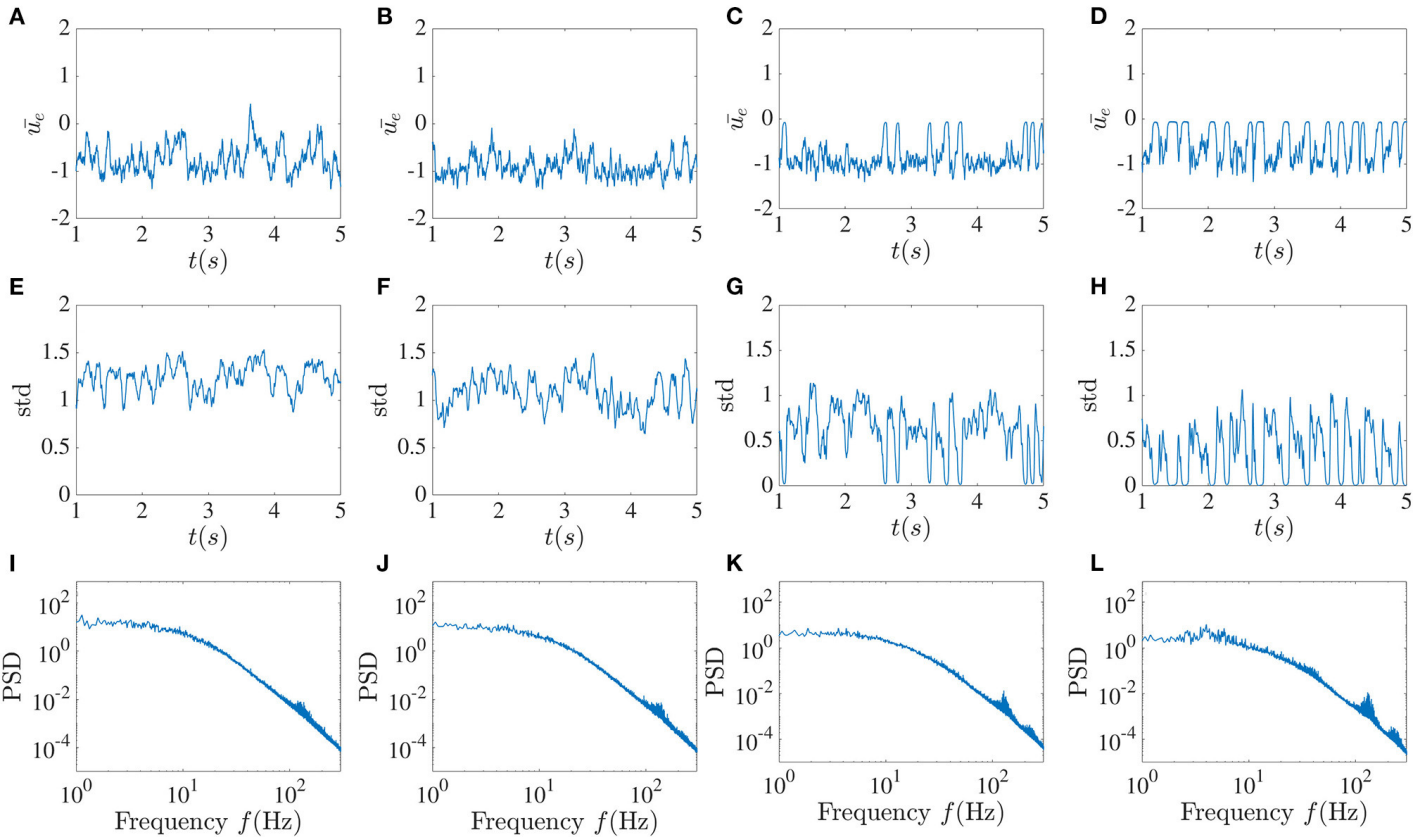
Higher global feedback strength causes activity dropouts. Mean **(A–D)** and standard deviation **(E–H)** of the excitatory sub-network activities, with the corresponding mean power spectrum **(I–L)** in the presence of global inhibitory feedback without noise. From left to right the global feedback strength κ equals −1, −2, −4, and −6 and in all cases, the global feedback time delay is *T* = 10 ms. Higher feedback strength causes more dropouts. As for the increased delay case, between dropouts the standard deviation increases.

We illustrate in Figure 5 the influence of the global feedback strength and assume that the local and global feedback time delays are fixed. As for the previous case where the delay was increased, we observe that increasing the strength of the global feedback also promotes synchrony between units. In the power spectra, similar to the case of increasing delay, the high-frequency components become more evident as the units are more synchronized. The enhanced standard deviation outside dropouts raises the possibility for spiking, a fact that will be confirmed below.

**Figure 6:**
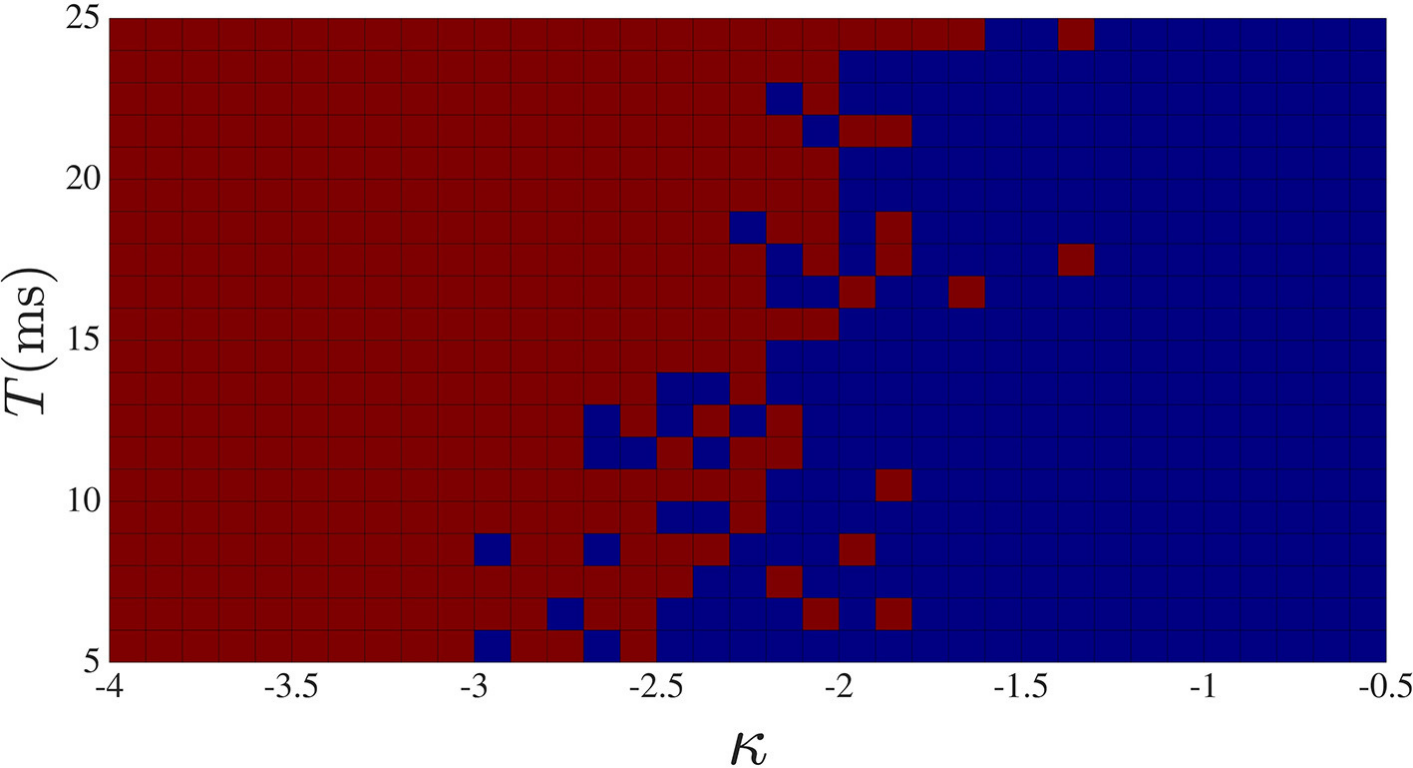
Activity drop-out phase diagram in κ-*T* space. Red squares correspond to the cases where at least one activity drop-out was observed during 35 s, and blue squares for the cases with no activity drop-out. Stronger and/or longer delay global feedback are seen to promote drop-outs.

So far, we have seen that either increasing the delay or the strength of the global feedback, the degree of complexity decreases. One difference between the two cases is that at large global feedback coupling, the dynamic will be stuck in a regime of high-frequency low-amplitude oscillation (not shown), while for large global feedback time delay, oscillation with low frequency is the dominant behavior of the sub-networks. The phase diagram for different κ and different global feedback time delay *T* is shown in Figure 6. For this computation, we counted the number of activity drop-outs during 35 s following a 2-s transient, repeating the simulation for different κ − *T* pair.

**Figure 7:**
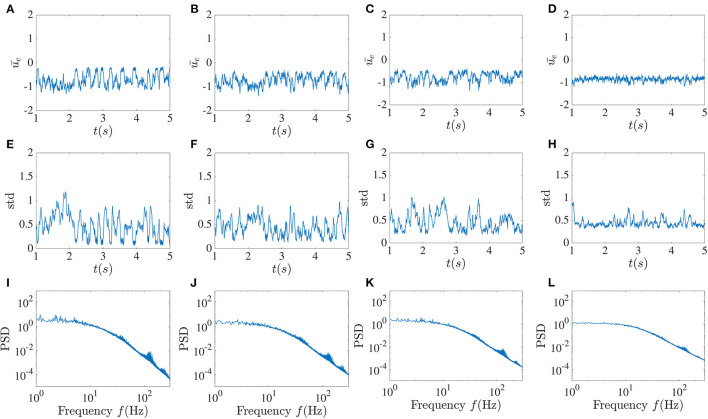
Noise suppresses activity dropouts. Mean activity **(A–D)**, standard deviation **(E–H)**, and power spectrum **(I–L)** averaged over the excitatory sub-network for increasing noise intensity *D* and fixed global inhibitory feedback with time delay *T* = 10 ms and strength κ = −5. From left to right, *D* = 10^−5^, 5 × 10^−5^, 10^−4^ and 5 × 10^−4^. The dropouts seen in Figure 4B are no longer seen, and the fluctuations of the standard deviation decrease at higher noise intensity.

The pattern of sudden low activities caused by the global feedback appears to be highly vulnerable as it can not be sustained in most cases, and asynchronous fluctuations may be reinstated. In Figure 7, these patterns are still found for small noise intensities, while the standard deviation fluctuations in these cases are more constrained. As the noise intensity increases, these dropouts are less likely to occur. It can be noticed that for significant noise intensity, the variations of the time-dependent standard deviation become more confined around 0.5; thus at higher noise, both the mean and the standard deviation seem to stabilize. This appears to be a form of noise-induced order from a chaotic state (Matsumoto and Tsuda, 1983). A simple picture here is that the noise in fact breaks up the synchronous periods and makes the dynamics more homogeneously asynchronous. Despite the decreasing occurrence of activity dropouts, the power spectrum still shows peaks around the high-frequency component, although they are reduced in size. The power spectrum at low frequency also becomes flatter as *D* increases, with a clear transition to a power-law regime at higher frequencies.

**Figure 8:**
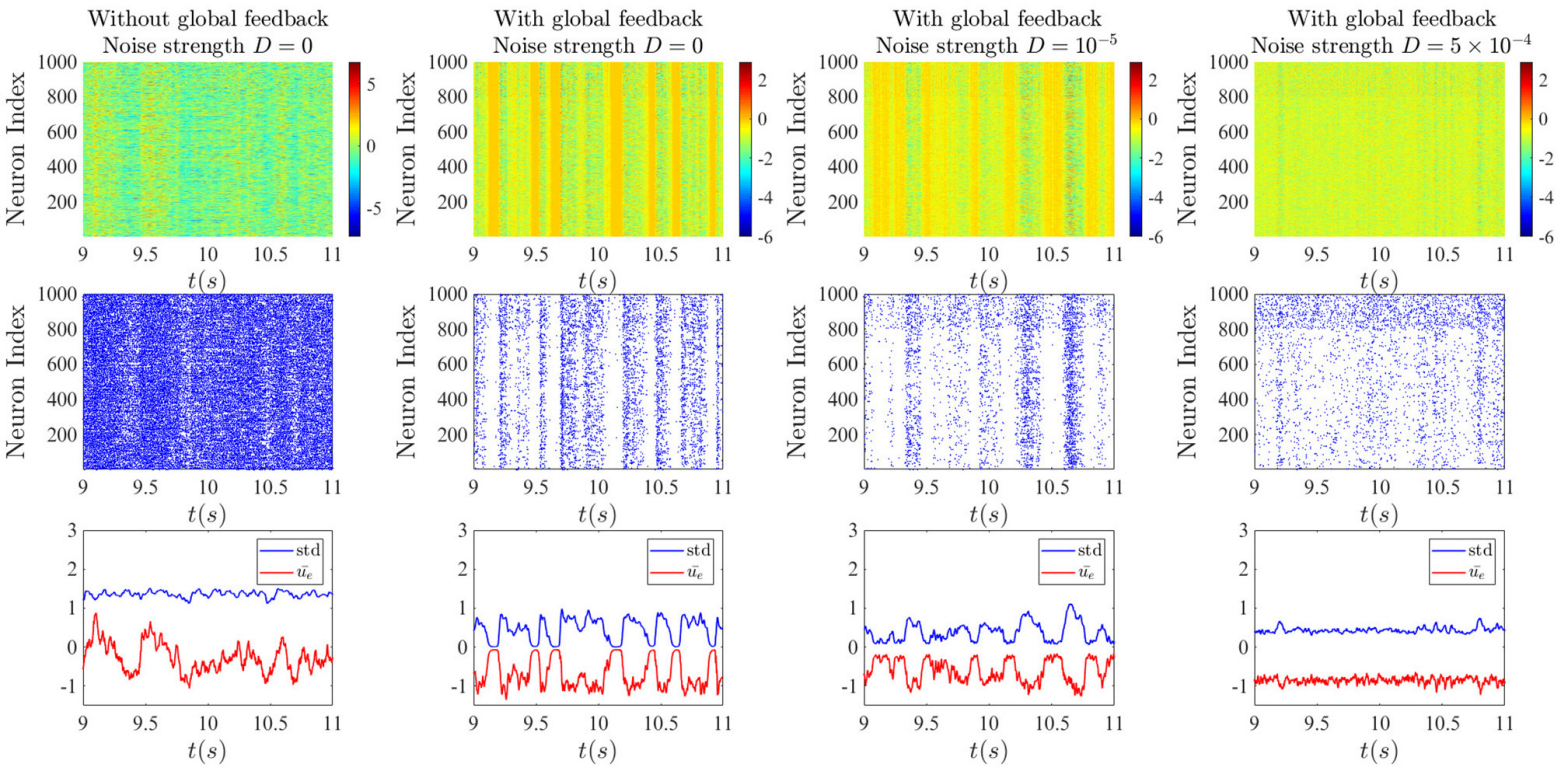
Effect of noise on chaotic global activity dropouts. **Top**: Raster plots of neuron activities from simulations of Equations (1a) and (1b). **Middle**: Raster plots of neuron spiking obtained by applying the Poisson spiking rule Equation (3) to simulations in the top row. **Bottom**: time-dependent mean and standard deviation of the activities of units in the excitatory sub-network. In the left column, the noise and the global feedback are set to zero. In the second column, the global inhibitory feedback is added, leading to the random occurrence of epochs of strong synchrony due to activity dropouts. The last two columns correspond to the cases with additive weak and strong noise on the dynamics of the units. The global inhibitory feedback delay *T* is 10 ms and its strength κ is −5. The top rows show that the inhibitory sub-network exhibits qualitatively the same behavior as the excitatory one, but with a slightly higher spiking rate.

From the raster plots in Figure 8, we can understand better the dynamics of all the neurons in the two different sub-networks for different cases. In the absence of the global feedback (left column), the mean network activity fluctuates more around the zero value, and it occurs with higher amplitude. In this case, high spiking activity can be observed, where this spiking activity of individual neurons is based on the assumption that firing follows an inhomogeneous Poisson process with the rate ϕ(*x*) (*x* is either *u* or *v*) and the probability of firing in an interval (*t, t* + *dt*) is given by (Rich et al., 2020):


(3) $$\begin{array}{l} p\left(x\right)=1-e^{-\phi \left(x\left(t\right)\right)dt}. \end{array}$$

By taking into account the global delayed feedback (three right columns), activity dropouts can be seen in yellow bars in the activity rasters at the top. With a strong enough global feedback coefficient, and sufficiently long delay, the amplitude of the fluctuations decreases and the mean of network activity shifts down to more negative values. This makes sense given that the global feedback is inhibitory. As a consequence, the network spiking activity decreases. We can see clearly that the dropouts are associated with epochs of high mean activity but low standard deviation of activity—hence the name “dropout.” For stronger noise intensity, the probability of dropouts decreases, resulting in slightly more widespread spiking activity.

In the middle row, it can be seen that for this set of parameters, spiking activity is slightly higher in the inhibitory sub-network compared to the excitatory sub-network, and there would rarely be a spike during an epoch of dropout. With the decreasing of the amplitude of the fluctuations of the standard deviation through increasing noise intensity, we see that somehow the spiking activity spreads out, especially in the inhibitory sub-network.

**Figure 9:**
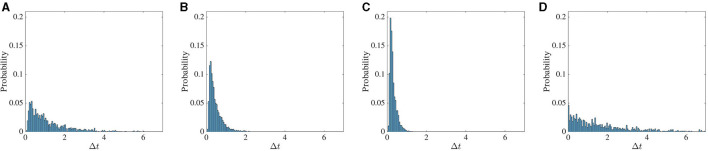
Frequency of chaotic activity dropouts increases with stronger feedback. The probability of intervals between successive low firing activity dropout events. In the first three columns **(A–C)**, the noise strength is *D* = 0 and the global feedback strength κ changes from −4 to −5 to −6. In **(D)**, separate noise terms, each with intensity *D* = 10^−5^, are added to the excitatory and inhibitory dynamics. In all cases the global feedback time delay *T* is 10ms.

The histogram of the time difference between the two dropout activities is shown in Figure 9. The statistic is calculated in the following way. We first take the arbitrary threshold value of 0.06 for the standard deviation. We store the data for a duration between the time the standard deviation falls below 0.06 and the time that it rises above 0.1. During this interval, we record the time corresponding to the minimum value of standard deviation. This process is repeated up to *t* = 1, 500 s.

First, we only varied the global feedback strength κ from −4 to −6, and the effect of noise was only considered in the last panel of Figure 9. Increasing the impact of global feedback on the dynamics coincides with the increase in the probability of these events in a shorter interval, and the statistic tends to be more Poissonian. Due to the noise, the fluctuation around the arbitrary threshold value increases and consequently, the time difference between these events decreases significantly. In general, however, greater noise levels tend to suppress dropout activity.

**Figure 10:**
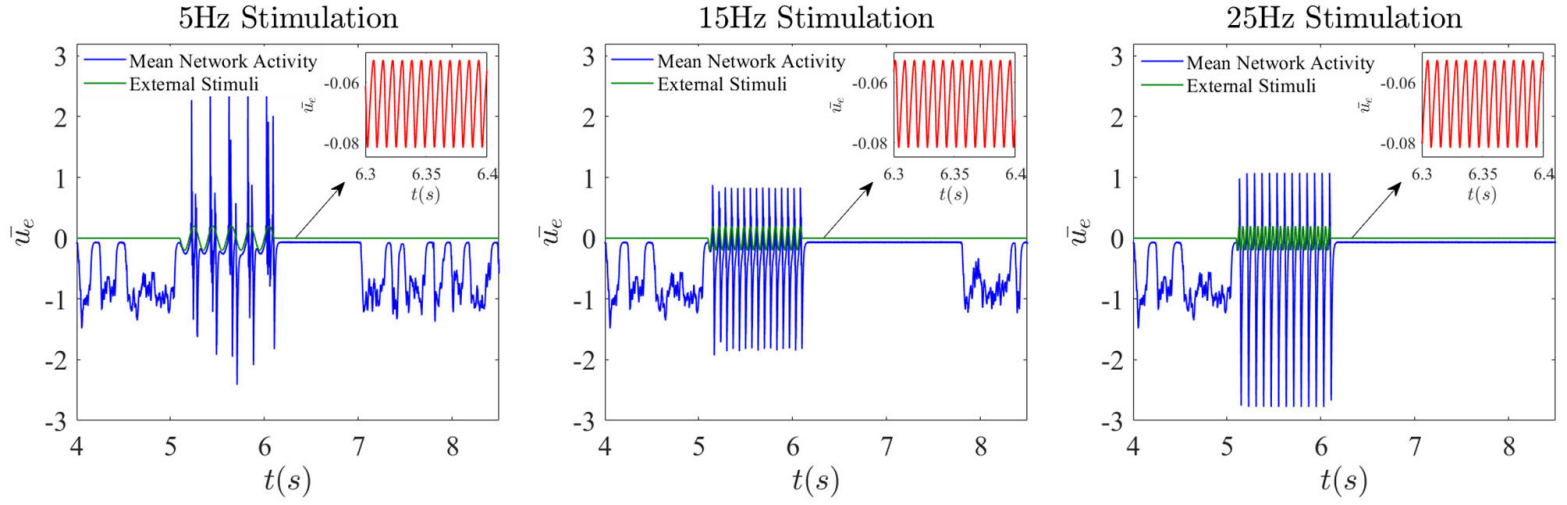
Frequency-dependent complexity collapse. Network dynamics when an external sinusoidal stimulation is activated for 1 s from *t* = 5.1 s until *t* = 6.1 s. Here the global feedback strength is κ = −5 and the corresponding time delay is 10 ms.

Externally applied stimuli can have a wide range of dynamical effects, including suppression of chaos, entrainment, etc (Rajan et al., 2010; Park et al., 2018). Of particular interest is the effect of external periodic stimuli (chosen here with an amplitude of 0.2) on the dynamics in the presence of dropout activities. In Figure 10, the noise is turned off, and only the sinusoidal external input with different frequencies is applied for a duration of 1 s. It can be seen that after a low-frequency stimulus such as 5 Hz ceases, the chaotic network activity prior to stimulation is replaced by a high frequency oscillation of 130 Hz. The duration of these simplified dynamics beyond the stimulation is found to vary as a function of stimulation frequency. For example, for a 15Hz stimulus, the duration elongates a little, but eventually the system recovers its natural dynamical properties. As seen third column in Figure 10, when the stimulation frequency is relatively higher, the chaotic dynamics may not be recovered at all, or at least not for a much longer time. Because timing and duration of stimulation are crucial in applications, how the network responds to stimulation appears highly complex, and a full investigation is beyond the scope of this paper.

**Figure 11:**
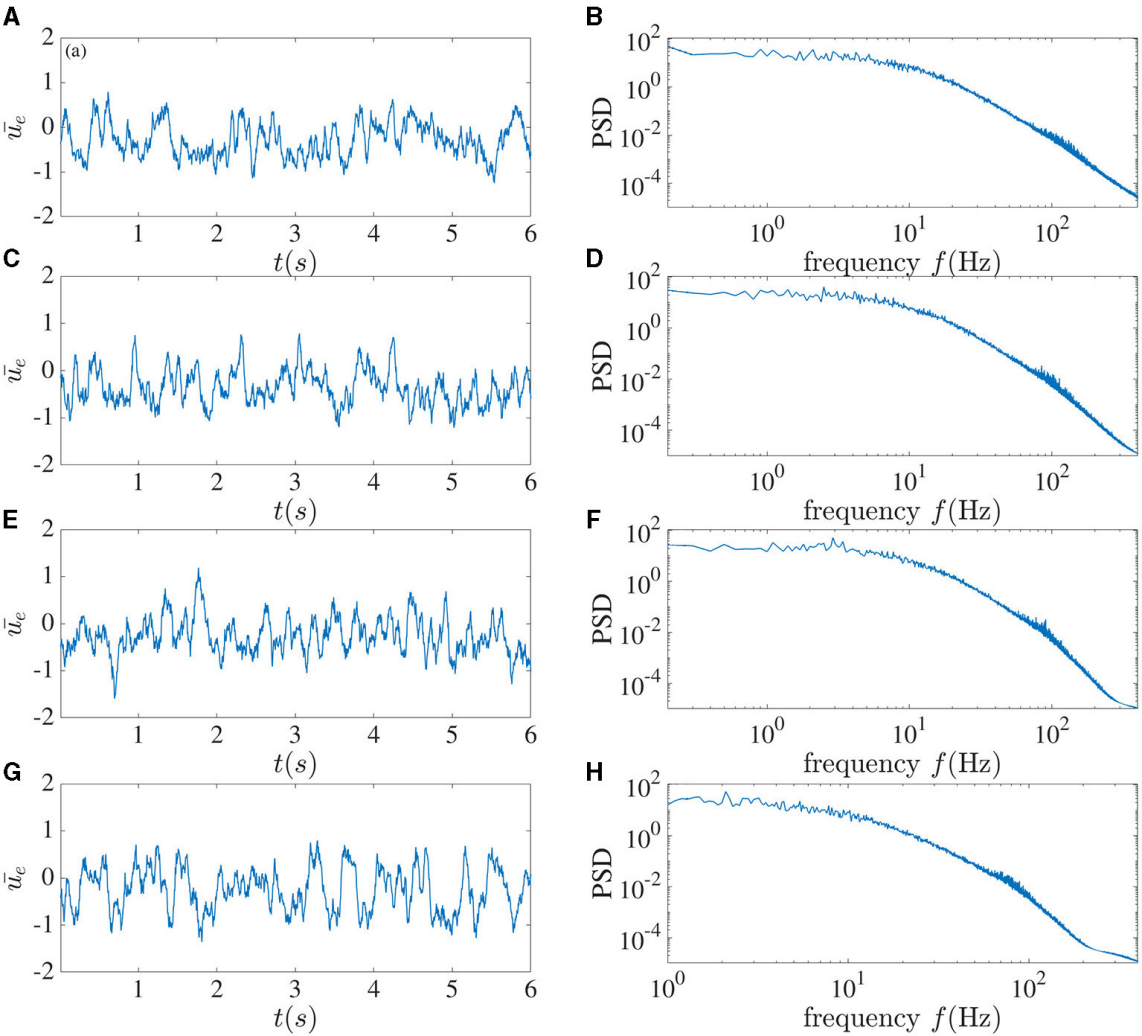
Broadening the local recurrent delay distribution has little effect in the absence of global delayed inhibition. Mean excitatory sub-network activity for different number of delays *M*. From top to bottom, *M* = 6 **(A,B)**, 11 **(C,D)**, 16 **(E,F)**, and 21 **(G,H)**. Here the global feedback and the noise are set to zero. The delays are confined to the interval [2 + 0.2(*M* − 1)] ms. No complexity collapse is seen, and the spectra are difficult to tell apart.

It has been shown (Tavakoli and Longtin, 2020) for many dynamical systems from lasers to biological feedback system that upon adding a sufficient number of delays to the dynamics, a transition from chaos to simpler behavior such as periodic motion, or even fixed-point behavior, can occur, provided that the range of delays is sufficiently broad. In Figure 11, we show the behavior of the network activity when multiple local delays are included. Here we set the noise to zero, as well as the periodic stimulation and the global feedback. We assume that the minimum delay is equal to 2 ms, and more delays added at Δτ = 0.2*ms* increments up to a maximum delay of [2 + 0.2(*M* − 1)] ms.

We carried out the simulation for *M* = 6, *M* = 11, *M* = 16, and *M* = 21. Figure 11 shows that, in contrast to the aforementioned delayed dynamical systems, the dynamical properties are not affected so drastically upon adding more delays. This is likely due to the fact that the local EI recurrent dynamics have sufficient intrinsic non-linearity to support chaos without relying on the delay. Our simulations for unrealistically large local delays (with large spacing between delays, and up to a largest delay of 242 ms for 21 delays) revealed no dropout activity or complexity collapse when there was no delayed global feedback (not shown).

**Figure 12:**
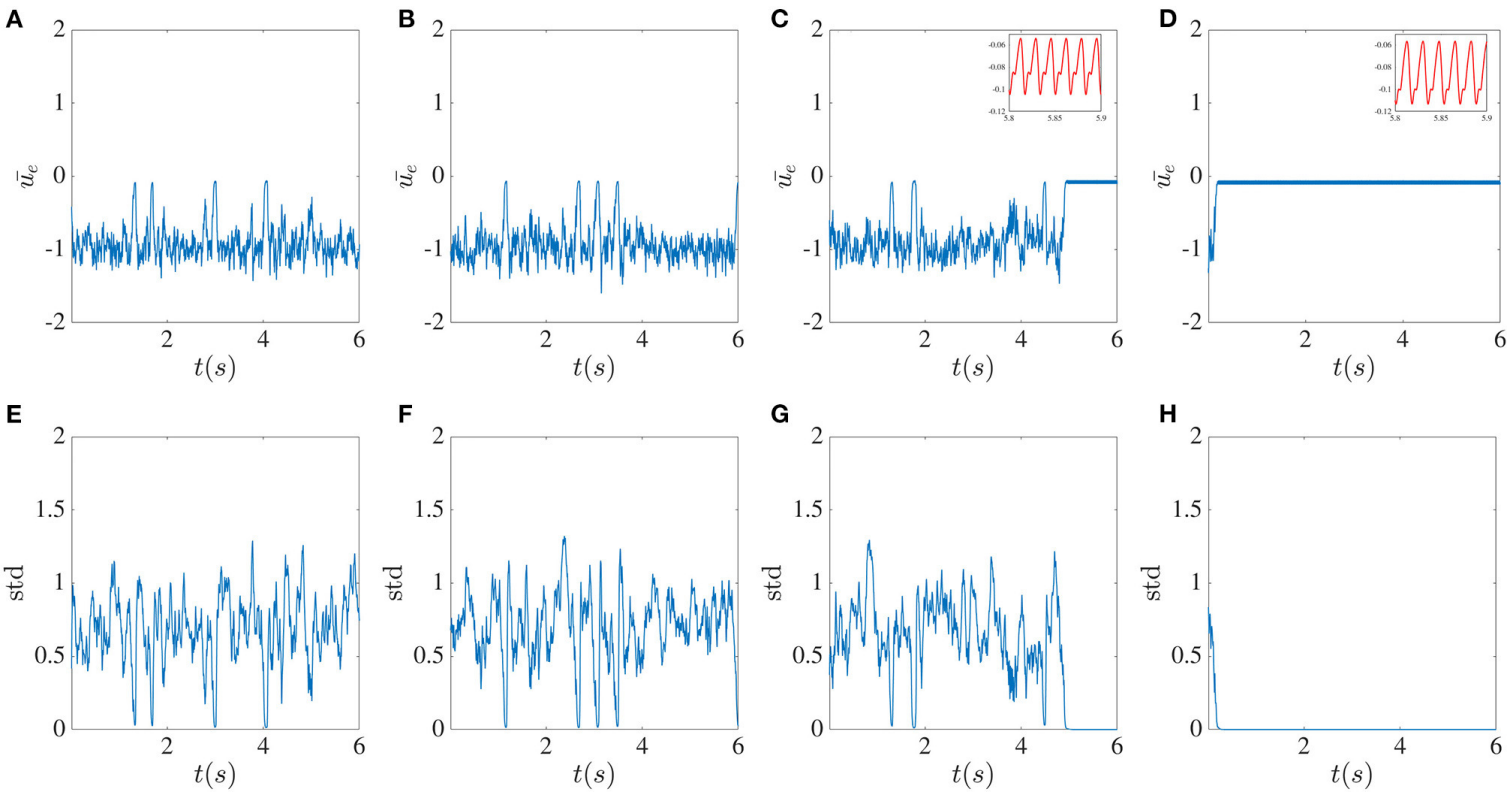
Broadening the local delay distribution initiates complexity collapse in the presence of global delayed feedback. Mean excitatory sub-network activity for different numbers *M* of local delays. From top to bottom, *M* = 6 **(A,E)**, 11 **(B,F)**, 16 **(C,G)**, and 21 **(D,H)**. The global feedback time delay *T* is 10*ms* and the feedback strength κ is −4. The delays are confined to the interval [2 + 0.2(*M* − 1)] ms. The CC occurs faster the stronger the delay is.

It is interesting that for a single delay case, as we saw in Figure 3, and for large enough delay, dynamics are simple oscillatory. However, the presence of smaller local delays makes the oscillatory dynamics chaotic. As we saw earlier, global feedback delay can decrease the degree of complexity in the chaotic dynamics. Therefore, we consider the dynamics of the network with multiple local delays in the presence of the global delayed feedback to see whether we can observe the complexity reduction with multiple delays. Parameters used in Figure 12 are the same as those used for Figure 11.

A key finding is that in the presence of both local recurrent delayed feedback and global inhibitory delayed feedback, the dynamics are significantly affected by the multiple local delay times. Indeed, Figure 12 reveals that, as the distribution of the time delays broadens, the system manifests transient chaos, which eventually converges to a periodic limit cycle attractor with the low amplitude oscillations. Hence, in the presence of a global inhibitory delayed feedback, the system exhibits CC; but it requires a longer delay inhibition to occur. The new feature with respect to the previously reported MDCC is that here the transition to simpler dynamical behavior involves transient chaos.

## 4. Discussion

We have focused on the properties of a rate-based neural network with a small number of short delays in the local sparsely connected EI recurrent circuitry, and how this is altered by a longer delay that acts globally through all-to-all feedback inhibition. Our goal was to investigate under which conditions, if any, a broadening of the local delay distribution can lead to a simplification of the chaotic dynamics seen for a single delay. By construction, the setup of this problem also allows a preliminary analysis of the effect of clusters of delays on local recurrent EI dynamics, although we have limited our study to two clusters, one of which contains only a single delay. But the means of these clusters are related by a factor of 2-3. Apart from being relevant to neural circuitry, the inclusion of the global feedback was found to be necessary to see CC in a chaotic EI neural network, if the local delays are not allowed to take on values that are too large.

Specifically, we first showed that an increase in the local time delay could lead to a drastic change in the deterministic dynamics. When this delay is unique and is increased from 2 ms to 10 ms, chaotic dynamics are abruptly replaced by regular periodic synchronized network firing (Figure 3). This is a first instance in which the complexity collapses in our network, although in a manner that does not rely on the inclusion of more delays (Tavakoli and Longtin, 2020); rather it appears to simply arise from a bifurcation when the single delay parameter is increased.

Adding a delayed global inhibitory feedback can however lead to different interesting phenomena. The main one, show in Figures 4, 5, features chaotic dynamics that exhibit sudden pulses which we have termed “activity dropouts.” This effect is more pronounced when the global feedback is strong or its delay is large. Interestingly it is also associated with a power law behavior of the mean activity over three orders of magnitude (only 2.5 orders are shown). These activities contain a high-frequency component that is embedded possibly an unstable orbit in the chaotic attractor due to the local time delay. This property becomes essential when other simplification factors are added to the system, such as increasing the number of local delayed interactions (Figure 12) or correlated input. While adding uncorrelated input, such as white noise, does not destroy this component completely, it helps maintain the activity's chaotic nature due to the recurrent local interaction (Figure 7). But paradoxically, additive noise on the dynamics also leads to a reduction in the size of the fluctuations in the time-varying standard deviation. This is a form of noise-induced order from a chaotic state first reported by Matsumoto and Tsuda (1983).

The activity dropouts are interesting because the global feedback makes the standard deviation (SD) of the solution on the attractor vary randomly (in fact, Poisson-distributed—see Figure 9). The mean of the activity is higher during the periods of low SD, yielding minimal spikes—thus the term “dropout.” During the periods of high SD, the mean activity is even lower, but the few cells that fluctuate the most are able to fire during the higher portions of these fluctuations, and their spikes drive the whole network activity. Note that the model does not explicitly run on spiking; the spikes are a derived quantity from Equation (3).

The more regularly aspects of the activity that involves dropouts is reminiscent of the stabilization of unstable periodic orbits using delayed feedback (Pyragas control), although the precise form of the global feedback used here differs from the one used in that chaos-control scheme. Nevertheless this global feedback may create or reveal an underlying slower rhythm embedded in the chaos and which becomes manifest as a lower frequency peak and its harmonics in the power spectra (see Figure 4L).

Complexity collapse in the sense of that in Tavakoli and Longtin (2020) does appear in our work through the broadening of the local delay distribution as seen in Figure 12; but for the parameter range where we found this effect, the global inhibitory feedback with longer delay must be present. It is possible that other regimes occur in which CC does not rely on the presence of this global feedback.

The novel behavior in Figure 8 is striking in that there is a temporally random appearance of epochs of dropouts. The time between these dropouts are reminiscent of up-states seen experimentally in neuroscience, and the dropouts as down states. This appears to be a novel deterministic behavior that is synchronized across the network, i.e., it is not a chimera. It survives the presence of moderate noise. There is a sense in which the global inhibitory feedback introduces longer time scales in the network dynamics - the stronger it is, the less power there is at low frequencies (Figure 5). This might share features and origins with the long time scales that arise from introducing population clusters—instead of delay clusters as done here - into EI networks in Litwin-Kumar and Doiron (2012).

Delayed inhibitory feedback has also been reported to elicit transitions between quasi-periodic partial synchronization and collective chaos (Pazó and Montbrió, 2016). Our dynamics here appear to differ from that scenario in that the collective behavior here is not periodic (our network also has E and I coupling). Another point of comparison is the work in Luccioli et al. (2019) where inhibition with long delay can bring on collective oscillations as we see here in Figures 4, 5; it remains to be seen whether a winner-take-all mechanism is at work in our system as reported there.

The final point of interest is the fact that the broadening of the local delay distribution brings on a collapse from chaos to simple (limit cycle) dynamics in a time inversely proportional to the width of that distribution (Figure 12). This is a form of transient chaos in neural networks (Zillmer et al., 2009) that relies here on delay clusters. It warrants a deeper investigation, especially of its dependence on the initial state of the network. It reflects special properties of the underlying attractor that are emphasized also in response to external inputs. Indeed we have uncovered a frequency-dependent silencing of the network activity, or frequency-dependent CC that can be temporary or even likely permanent, depending on the frequency. It is a different form of persistence from stimulation reported in Park et al. (2018); in particular, the silencing time seems to depend on the timing of when the stimulus is applied (not shown). This will be investigated elsewhere. This may bear on the reaction of the activity of a neural network with delay clusters to extraneous rhythms or artificial stimulation.

## Data Availability Statement

The raw data supporting the conclusions of this article will be made available by the authors under request, without undue reservation.

## Author Contributions

ST and AL conceived the principle idea of the work and structured the manuscript. ST carried out the numerical simulation. All authors have written the manuscript.

## Funding

This work was supported by Natural Sciences and Engineering Research Council of Canada under Grant No. RGPIN/06204-2014.

## Conflict of Interest

The authors declare that the research was conducted in the absence of any commercial or financial relationships that could be construed as a potential conflict of interest.

## Publisher's Note

All claims expressed in this article are solely those of the authors and do not necessarily represent those of their affiliated organizations, or those of the publisher, the editors and the reviewers. Any product that may be evaluated in this article, or claim that may be made by its manufacturer, is not guaranteed or endorsed by the publisher.
